# Step-wise elimination of α-mitochondrial nucleoids and mitochondrial structure as a basis for the strict uniparental inheritance in *Cryptococcus neoformans*

**DOI:** 10.1038/s41598-020-59277-9

**Published:** 2020-02-12

**Authors:** Yoshiki Nishimura, Toshiharu Shikanai, Susumu Kawamoto, Akio Toh-e

**Affiliations:** 1 Department of Botany, Kyoto University, Kita-Shirakawa, Oiwake-cho, Kyoto 606-8502 Japan; 2 Division of Clinical Research, Medical Mycology Research Center, Chiba University, 1-8-1 Inohana, Chuo-ku, Chiba 260-8673 Japan

**Keywords:** Mitochondria, Fungal genetics, Fungal genetics

## Abstract

In most sexual eukaryotes, mitochondrial (mt) DNA is uniparentally inherited, although the detailed mechanisms underlying this phenomenon remain controversial. The most widely accepted explanations include the autophagic elimination of paternal mitochondria in the fertilized eggs and the active degradation of paternal mitochondrial DNA. To decode the precise program for the uniparental inheritance, we focused on *Cryptococcus neoformans* as a model system, in which mtDNA is inherited only from the **a**-parent, although gametes of **a-** and α-cells are of equal size and contribute equal amounts of mtDNA to the zygote. In this research, the process of preferential elimination of the mitochondria contributed by the α-parent (α-mitochondria) was studied by fluorescence microscopy and single cell analysis using optical tweezers, which revealed that α-mitochondria are preferentially reduced by the following three steps: (1) preferential reduction of α-mitochondrial (mt) nucleoids and α-mtDNA, (2) degradation of the α-mitochondrial structure and (3) proliferation of remaining mt nucleoids during the zygote development. Furthermore, *AUTOPHAGY RELATED GENE* (*ATG*) 8 and the gene encoding mitochondrial endonuclease G (*NUC*1) were disrupted, and the effects of their disruption on the uniparental inheritance were scrutinized. Disruption of *ATG8 (ATG7)* and *NUC1* did not have severe effects on the uniparental inheritance, but microscopic examination revealed that α-mitochondria lacking mt nucleoids persisted in Δ*atg8* zygotes, indicating that autophagy is not critical for the uniparental inheritance *per se* but is responsible for the clearance of mitochondrial structures after the reduction of α-mt nucleoids.

## Introduction

Mitochondria and chloroplasts are organelles responsible for ATP production via respiration and photosynthesis, respectively. In addition, these organelles function as centers for diverse cellular processes, including lipid biosynthesis, amino acid metabolism, and cell death. Mitochondria and chloroplasts contain their own genomes, which are thought to be vestiges of their bacterial ancestors^1^. Unlike nuclear DNA, one or two copies of which are present per cell, hundreds to thousands of copies of mitochondrial (mt) DNA are present in individual cells. Usually, each of the cells maintains only one genotype of mtDNA (homoplasmy), which is inherited from the maternal parent. Maternal inheritance of mitochondrial (chloroplast) genomes is commonly observed in sexual eukaryotes, including animals, plants, fungi, and protists^2–4^.

Uniparental/maternal inheritance was once considered to be a result of the dilution of the paternal contribution because the paternal gametes (sperm) are much smaller than maternal gametes (eggs) and contribute a limited amount of cytoplasm to the progeny^5^. However, this simple dilution model has been challenged by many findings^6^. Currently, various mechanisms have been proposed to explain the active elimination of paternal mitochondrial or chloroplast genomes, including specific nucleases^7–9^, physical exclusion of paternal organelles upon gametogenesis or fertilization^7,10^, and the most extensively debated proposed mechanism: autophagy^11–15^.

It has been reported that sperm mitochondria are sequestered by autophagosomes and delivered to lysosomes for further degradation in fertilized eggs^11,15^. RNAi-mediated down-regulation of a gene homologous to autophagy-related protein (*ATG*) 8, which is necessary for autophagosome formation, caused longer retention of paternal mtDNA in fertilized eggs of *Caenorhabditis elegans*^11^. Based on these results, it has been proposed that maternal inheritance is regulated by autophagy. In the cross between the *C*. *elegans* RNAi strains, however, most of the eggs did not hatch or died prematurely^11^, raising the possibility that the role of autophagy is so critical in the early embryogenesis that the defective autophagy might have impeded degradation of paternal mtDNA as a secondary effect. Indeed, the occurrence of normal uniparental inheritance in autophagy-defective mutants has been reported in various organisms, including animals, fungi, and green algae^12–14,16^.

Meanwhile, an intriguing example of uniparental inheritance occurs in the unicellular green alga *Chlamydomonas reinhardtii*, which produces identically sized (isogamous) female (mating type plus) and male (mating type minus) gametes. It is now believed that the uniparental inheritance of chloroplast (cp) DNA is achieved via the rapid digestion of paternal cp nucleoids (cpDNA-protein complexes)^17^, triggered by gamete-specific homeotic genes^18^. Also, active digestion of uniparental mt nucleoids (mtDNA-protein complexes) has been observed in the slime mold *Physarum polycephalum*^19^ and in sperm mitochondria of medaka fish *Oryzias latipes*^20^, and notably, these events occurred prior to the destruction of mitochondria. Recently, it has been reported that mitochondrial endonuclease G relocates from the intermembrane space to the matrix after fertilization to eliminate paternal mtDNA. Endonuclease G has been reported to be involved in the elimination of mtDNA during sperm development in *Drosophila melanogaster*^7^. Currently, endonuclease G is an interesting candidate for a driving force for the degradation of paternal mt nucleoids, but although its down-regulation caused embryo lethality in *C*. *elegance*^8^, in *D*. *melanogaster*, there is a secondary mechanism that compensates for defects of endonuclease G by physically removing paternal mitochondria from mature sperm^7^.

Various mechanisms have been proposed to explain the uniparental inheritance thus far. To decipher the precise mechanism for the uniparental inheritance of mitochondria, we focused on a basidiomycete yeast, *Cryptococcus neoformans*. *C*. *neoformans* is a major human and animal pathogen known to cause severe meningoencephalitis, particularly in immunocompromised individuals such as those with HIV/AIDS^21^. From the standpoint of basic biology, *C*. *neoformans* is an attractive eukaryotic model organism to analyze the basic mechanisms underlying the reproduction and the uniparental inheritance of mtDNA^22^, because of its short sexual life cycle, an efficient transformation protocol, genetic tractability, and abundant genome information^23^. *C*. *neoformans* has a bipolar mating system in which the mating type (namely, either **a-** or α-cells) is determined by a single genetic locus (the *MAT* locus). The mating process, which is governed by a complex genetic network, has been elucidated^24,25^. Importantly, it has been found that mtDNA is almost exclusively inherited from the **a**-parent, despite the almost equal sizes of the **a-** and α-cells^26–29^. The uniparental inheritance of mtDNA has been studied genetically, but the behavior of mitochondria and mt nucleoids has not been monitored microscopically, and the function and the precise role of autophagy remain unclear. Accordingly, here we aimed to visualize and monitor the behaviors of mt nucleoids and mitochondrial structures during fertilization in *C*. *neoformans*.

## Results

### Preferential reduction of α-mt nucleoids in zygotes

**Figure 1:**
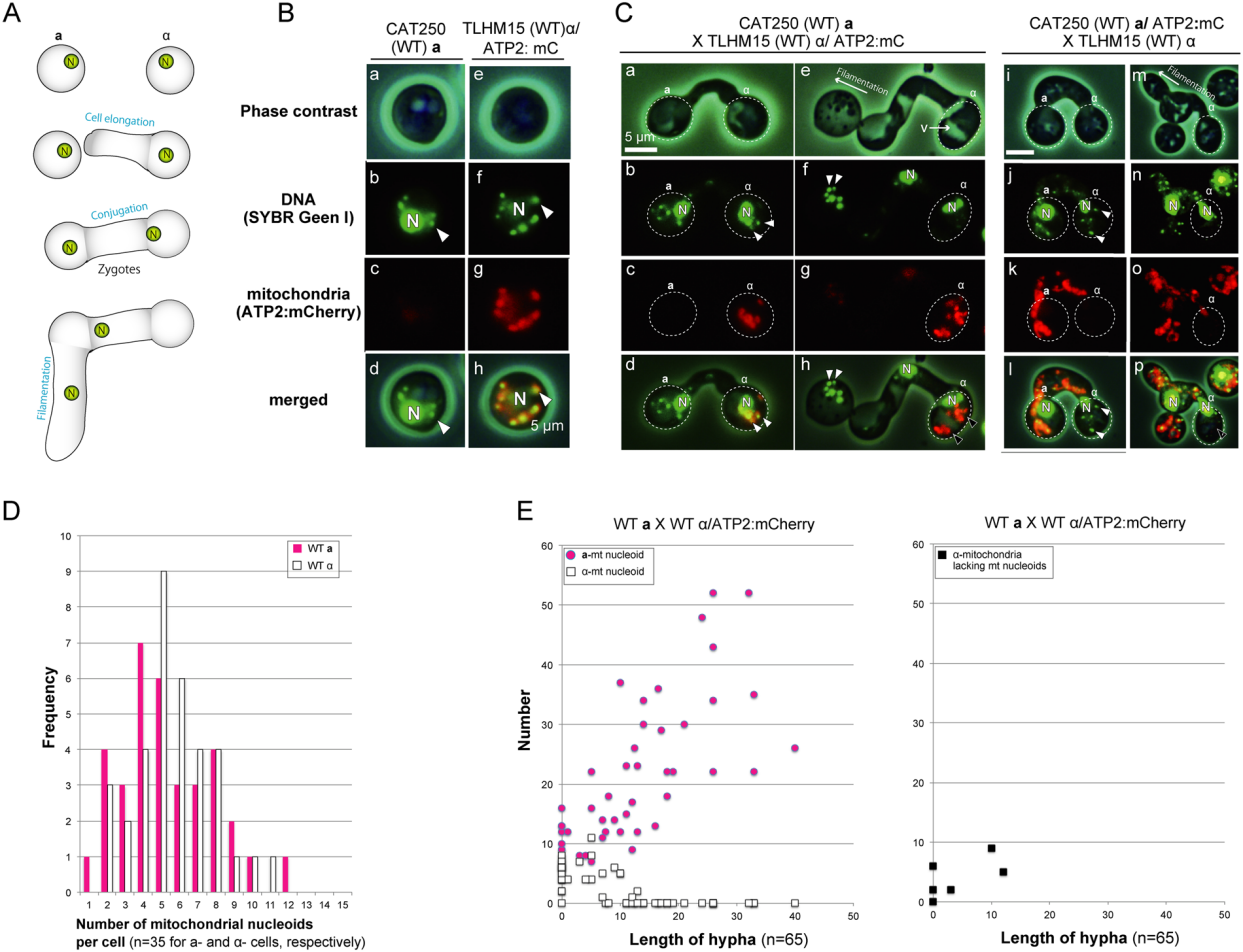
The sexual reproduction and preferential reduction of α-mitochondrial nucleoids in early zygotes of *C*. *neoformans*. (**A**) A schematic presentation of the mating process in *C*. *neoformans*. **a-** and α-haploid yeast cells secrete peptide pheromones that trigger **a**–α cell fusion under nutrient-limiting conditions. After fusion, zygotes switch to filamentous growth. (**B**) Detection of mt nucleoids in CAT250**a** (a-d) and TLHM15α/ATP2:mCherry (TLHM15α transformed with *ATP2*:mCherry) (e–h). Haploid cells were stained with SYBR Green I (b,f). *ATP2*:mCherry was used to visualize α-mitochondria (g). Phase contrast (a,e), SYBR Green I (b,f), mCherry signals (c,g), and merged images (d,h) are shown. (**C**) The behavior of mt nucleoids in the cross CAT250 (WT) a X TLHM15 (WT) α/ATP2:mCherry (a–h: α-mitochondria were labeled by mCherry) and the reciprocal cross CAT250 (WT) **a**/ATP2:mCherry X TLHM15 (WT) α (i-m: **a**-mitochondria were labeled by mCherry). Mt nucleoids were detected in the early zygotes in both **a**-and α-mitochondria (a–e, i–l: white arrowheads). However, in zygotes with developing hypha, α-mitochondria lacking mt-nucleoids were observed [black arrowheads in h and p], whereas **a-**mt nucleoids proliferated (h,p). N: cell nucleus. (**D**) A histogram that shows the number of mt nucleoids in haploid cells (WT **a** (magenta) and WT α (white)). The number of haploid cells analyzed was 40 each for **a** and α. (**E**) The number of **a**-(magenta squares) and α- (white squares) mt nucleoids (and α-mitochondria lacking mt nucleoids (black squares)) was plotted against the length of hypha in zygotes (WT **a** X WT α/ATP2:mCherry). The number of zygotes analyzed was 65.

In *C*. *neoformans*, sexual reproduction commences when yeast cells of opposite mating types (**a** and α) are mixed under nutrient-depleted conditions. Mating involves the formation of conjugation tubes in response to pheromones. Cytological studies show unidirectional migration of the nucleus from the α-cell to the **a**-cell during conjugation. The cell fusion product contains nuclear genomes of both parents and is equivalent to the zygote of plants and animals. The two parental nuclei in the zygote remain unfused until the basidium is formed. A dikaryotic mating hypha emerges from the zygote on the side of the original parental **a**-cell, followed by septation between the cell and zygote^30^ (Fig. 1A).

To monitor the precise behavior of **a-** and α-mtDNA in zygotes, it was necessary to visualize mt nucleoids (mtDNA-protein complexes which are thought to be the units of various processes, including mtDNA replication, repair, recombination, transcription, and inheritance)^31^. To this end, living *C*. *neoformans* cells were stained with the highly permeating dsDNA-specific fluorochrome SYBR Green I, which resulted in clear visualization of 5~10 fluorescent dots (~0.1–0.5 µm) per cell. α- or **a**-mitochondria were labeled with mCherry by linking the *mCherry* gene to the *ATP2* gene, which encodes the β-subunit of mitochondrial ATP synthase. The SYBR Green I-stained fluorescent dots precisely overlapped with the mCherry signal, confirming that they were mt nucleoids (Fig. 1B).

Early zygotes just after conjugation could be identified by their dumbbell shape and the absence of dikaryotic hyphae. In these zygotes, both α- and **a**-mitochondria showed yellow dots stained by SYBR Green I, representing mt nucleoids. However, as zygotic development progressed and upon the onset of filamentation, the α-mt nucleoid signals disappeared, whereas the structure of α-mitochondria, represented by the mCherry signal, remained apparently intact (Fig. 1C).

The numbers of **a**- and α-mt nucleoids in haploid cells and in the developing zygotes were quantified (Fig. 1D). The number of mt nucleoids was almost identical between **a**- and α-haploid cells (a: 5.625 ± 2.55 per cell and α:5.78 ± 2.20 per cell). As for mt nucleoids in zygotes, in our current experimental conditions the mating efficiency was low (~5–10%) and it was difficult to synchronize the zygote maturation process. So the number of mt nucleoids in zygotes (and mitochondria lacking mt nucleoids) was plotted versus the length of elongating hyphae, which is likely to reflect the time after zygote formation (Fig. 1E). As the elongation of hyphae proceeded, the number of α-mt nucleoids declined. In contrast, the number of **a**-mt nucleoids increased drastically.

To confirm these cytological observations, single early zygotes retaining α-mt nucleoids (E) and later zygotes lacking α-mt nucleoids were selectively trapped and harvested into PCR tubes using optical tweezers (Fig. S2, Video S1)^20^. The use of optical tweezers is particularly useful for analyzing *Cryptococcus* conjugation because synchronization of the mating reaction is difficult and the cell population is highly heterogeneous.

**Figure 2:**
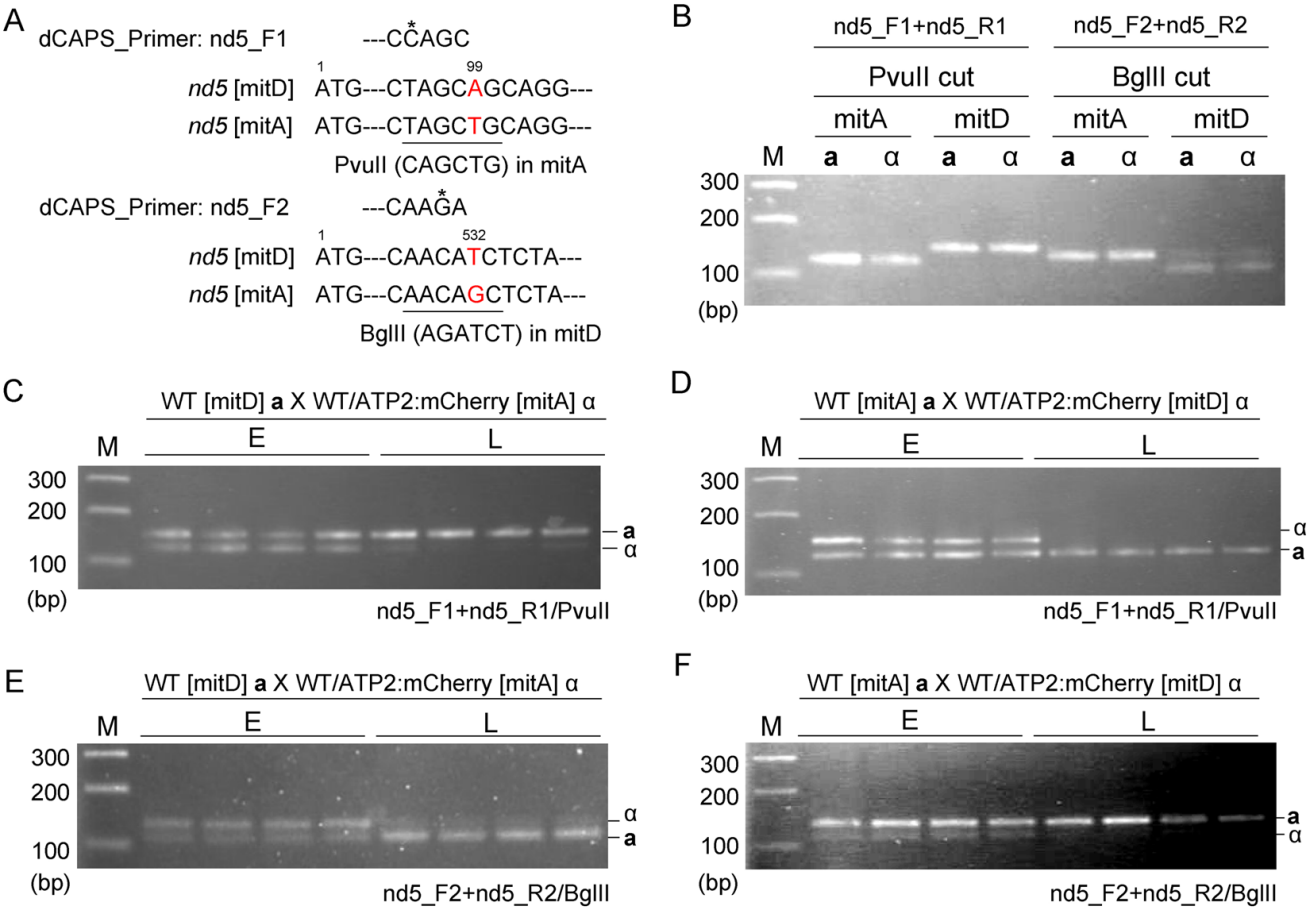
Relative reduction of α-mtDNA revealed by single-cell analysis using optical tweezers. Single zygotes at the early stage (E: early zygotes with α-mt nucleoids) and the later stage (L: later zygotes lacking α-mt nucleoids and with dikaryotic hyphae) were selectively trapped and harvested into PCR tubes for single-cell analysis. (**A**) dCAPS primers used to distinguish mitD and mitA sequences. dCAPS primer nd5_F1 creates a PvuII site only in mitA, whereas dCAPS primer nd5_F2 creates a BglII site only in mtD. (**B**) Total DNA was extracted from X258-1A **a** (WT [mitA]**a**), X258-1D α (WT [mitA]α), CAT250**a**/ATP2:mCherry (WT/ATP2:mCherry [mitD]**a**), and TLHM15α/ATP2:mCherry (WT/ATP2:mCherry [mitD]α), and digested with PvuII and BglII. (**C**) Single zygotes (WT [mitD]**a** X WT/mC [mitA]α) and (**D**) (WT[mitA] **a** X WT/mC [mitD]) were isolated using optical tweezers and analyzed by nested PCR (1^st^: nd5_F0 + nd5_R0, 2^nd^: nd5_F1 + nd5_R1). The PCR products were treated with PvuII, which cuts only the mitA sequence. (**E**) Single zygotes (WT[mitD] **a** X WT/ATP2:mCherry [mitA] α) and (WT [mitA] **a** X WT/ATP2:mCherry [mitD] α) were isolated and analyzed by nested PCR (1^st^: nd5_F0 + nd5_R0, 2^nd^: nd5_F2 + nd5_R2). The PCR products were treated with BglII, which cuts the mitD sequence. Typical results of three independent experiments (using 48 zygotes in total) are shown.

To distinguish between **a-** and α-mtDNA by PCR, strains with two different mitochondrial genotypes (mitD and mitA) were used for crosses, as per previous reports^29,32,33^. Nested PCR was used to detect the small amount of mtDNA contained in a single cell. Since PCR tends to amplify smaller fragments more efficiently, dCAPS primers were designed to circumvent this bias by detecting a single-nucleotide polymorphism between mitD and mitA (Fig. 2).

CAT1072 **a** [mitA] (A**a**) was crossed with TLHM15α [mitD] harboring *ATP2*:mCherry (Dα). In early zygotes, both mitA and mitD sequences were detected. However, only the mitA sequence (from the **a**-parent) was detected in later zygotes. These results were confirmed in the reciprocal cross (D**a** X Aα) (Fig. 2). Taken together, these results indicate that α-derived mtDNA molecules were degraded along with the disappearance of α-mt nucleoids in the early zygotes, prior to the elimination of the α-mitochondrial structure (Fig. 1).

### Preferential clearance of α-mitochondrial membrane structures in zygotes

**Figure 3:**
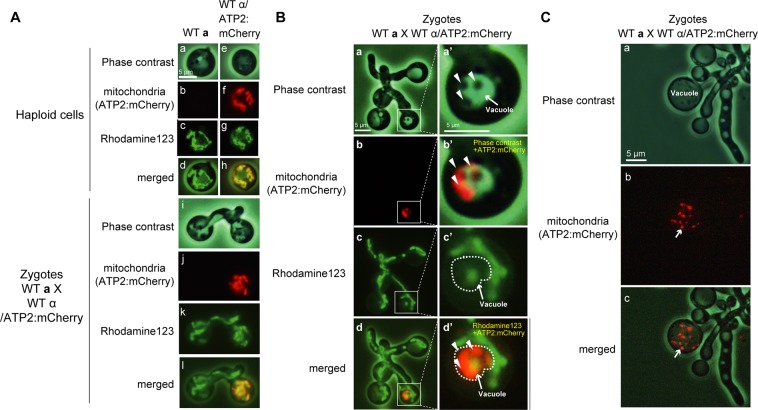
α-mitochondria are incorporated into vacuoles. (**A**) WT **a** haploid cells (a–d), WTα/ATP2:mCherry haploid cells (e–h), and zygotes (i–l) were stained with Rhodamine 123. *ATP2*:mCherry was used to track the behavior of α-mitochondria during zygote development. At this stage, both a- and α-mitochondria were stained with Rhodamine 123, indicating that they maintained the membrane potential. (**B**) Phase contrast (a), ATP2:mCherry (b), Rhodamine 123 signal (c) and merged images (d) of a zygote undergoing filamentation. (a’)–(d’) are magnified images of the area indicated by an open square in (a)–(d), respectively. b’ is a merged image showing phase contrast and ATP2:mCherry signal and d’ is a merged image showing Rhodamine 123 and ATP2:mCherry signals. During filamentation of zygotes, vacuoles are formed and α-mitochondria are engulfed in vacuoles. The engulfed α-mitochondria retained ATP2:mCherry signals but lost Rhodamine 123 signals, indicating loss of membrane potential (b’,c’,d’). (**C**) Phase contrast (a), *ATP2*:mCherry (b), and merged images (c) captured from Video S2. In the vacuole, α-mitochondria labeled with mCherry (b,c: white arrow) were accumulated as particles showing Brownian motion, indicating the specific incorporation and degradation of α-mitochondria during the zygote development.

The behavior of **a**- and α-mitochondrial structures during the zygote maturation was further monitored using Rhodamine 123, which visualizes mitochondrial membranes depending on the inner membrane potential, and whose signal is lost in depolarized mitochondria. Wild-type (WT) **a**-strain (CAT250**a**) was crossed with an α-strain (TLHM15α/mC) expressing *ATP2*:mCherry (Fig. 3). In the dumbbell-shaped early zygotes, both **a**- and α-mitochondria were stained by Rhodamine 123, indicating that **a-** and α-mitochondria maintained their membrane potential at this stage.

During the hyphal elongation, the development of vacuoles occurred. In some zygotes, small particles showing Brownian motion were observed within the vacuoles (Fig. 3; Video S2). The particles showed an mCherry signal (Fig. 3B,C, Video S2) and were only faintly stained with Rhodamine 123 (Fig. 3C,D), suggesting that they might be α-mitochondria undergoing degradation after having lost their membrane potential. Such zygotes with mCherry-labelled particles in vacuoles were extremely rare (1 in 32 zygotes: ~3.1%), probably due to their rapid degradation in vacuoles.

We also tried to visualize vacuoles by using a vacuole marker (MDY-64) (Fig. S2). In haploid cells, vacuoles were clearly observed frequently in the center of cells, and almost all mCherry-labelled mitochondria were detected outside of vacuoles. In zygotes, membrane structures were more complicated. When α-mitochondria were labeled with mCherry (WT **a** X WT α/ATP2:mCherry), mCherry-labelled α-mitochondria were surrounded by MDY-64-stained membranes in 2 out of 29 zygotes (~6.9%), suggesting that they might be engulfed by vacuoles (Fig. S2). In the reciprocal cross (WT **a**/ATP2:mCherry X WT α), proliferation of mCherry-labelled **a**-mitochondria was observed outside of vacuoles (Fig. S2). These observations indicated that α-mitochondria may be selectively engulfed in vacuoles, depolarized, and degraded. In yeast, the final destination of damaged mitochondria during autophagy (mitophagy) can be monitored using GFP fused to the mitochondrial outer membrane protein Om45^34^. Om45:GFP is transferred into vacuoles, causing the vacuoles to fluoresce, similarly to the behavior of α-mitochondria (Fig. 3, Video S2 and Fig. S2).

Next, we tried to monitor mtDNA synthesis by *in vivo* incorporation of the thymidine analogue 5-ethynyl-2′-deoxyuridine (EdU) during the zygote development. EdU incorporation was visualized in fixed cells with Alexa Fluor 488 using copper click chemistry, following a protocol reported for *Saccharomyces cerevisiae*^35^. **a**-and α-haploid cells were transferred to hay cube agar plates supplemented with EdU and allowed to form zygotes. When the cells were fixed, the image of mt nucleoids became blurry despite our efforts to find optimal fixatives and conditions, probably due to their extremely fragile nature. On the other hand, the ATP2:mCherry signal was clearly retained, so we decided to take advantage of ATP2:mCherry fluorescence to identify the mitochondrial EdU signal. To accomplish this, WT**a**/ATP2:mCherry was crossed with WTα. After the click reaction, EdU signals in the cell nucleus and cytoplasm were detected in ~70% of cells, and the cytoplasmic EdU signals were found to overlap with ATP2:mCherry, indicating that they reflected mtDNA synthesis (Fig. S3). The fluorescence intensity of mitochondrial EdU signals was quantified using ImageJ after the area of the cell nuclei (marked by DAPI staining) was subtracted from each of the images, using the image calculator of ImageJ. The mitochondrial EdU signals were almost identical between **a**- and α-haploid cells (Fig. S3B). Also, no significant difference was detected between **a**- and α-mitochondria in early zygotes without hypha, which indicates that the rate of mtDNA synthesis may not be drastically differentiated between **a**- and α-mitochondria in haploid cells (gametes) and early zygotes, although it should be noted that subtle differences in zygotes might have been masked due to the incorporation of EdU before zygote formation. Meanwhile, clear EdU signals were detected in **a**-mitochondria in elongating hyphae, and the EdU signals increased in zygotes with the elongation of hypha (Fig. S3C), supporting the conclusion that the increase of the number of **a**-mt nucleoids is probably accompanied by mtDNA synthesis.

Mitochondrial EdU signals in the parental **a**-cell areas and α-cell areas were also measured (Fig. 3D). The mitochondrial EdU signal remained rather constant in **a**-cell areas. In contrast, a gradual reduction of mitochondial EdU signal was observed in α-cell areas, which might have been caused by the preferential reduction of α-mt nucleoids/mtDNA in zygotes or by the preferential reduction of α-mtDNA synthesis.

### Autophagy is not required for the uniparental inheritance of mtDNA *per se*, but is responsible for the destruction of the α-mitochondrial structure after the reduction of mt nucleoids

To assess the function of autophagy in the uniparental inheritance, an *ATG8* homolog (CNA07930) in *C*. *neoformans* was disrupted by overlap PCR. The mtDNA inheritance was assessed in the crosses with Δ*atg8* as *MAT****a*** or *MAT*α (Table 1). As a positive control, the WT cross was performed at high temperature (33 °C), which partially disturbed mtDNA inheritance.

**Table 1:** Genetic analysis of the uniparental inheritance of mtDNA in Δ*atg8*, Δ*atg7* and Δ*nuc1* backgrounds.

Cross (a × α)	D:A	Leakage from α (%)
WT CAT250a [mitD] × WT X258-1Dα [mitA]	48 2	4.0
WT X258-1Aa [mitA] × WT TLHM15α [mitD]	3 56	5.1
WT CAT250a [mitD] × WT X258-1Dα [mitA] (33 °C for 2 day)	43 16	27.1
*MAT*a Δ*atg8*[mitD] × *MAT*α Δ*atg8* [mitA]	84 8	8.7
*MAT*a Δ*atg8*[mitA] × *MAT*α Δ*atg8* [mitD]	4 48	7.7
*MAT*a Δ*atg8*[mitD] × WT M15α [mitA]	37 2	5.1
WT CAT250a [mitD] × *MAT*α Δ*atg8* [mitA]	92 8	8.0
*MAT*a Δ*atg7* [mitA} × *MAT*α Δ*atg7* [mitD]	4 43	8.5
*MAT*a Δ*nuc1* [mitD] × *MAT*α Δ*nuc1* [mitA]	45 2	4.3
*MAT*a Δ*atg8*Δ*nuc1*[mitD] × MATα Δ*atg8*Δ*nuc1*[mitA]	95 10	9.5

Uniparental inheritance of mtDNA in *C*. *neoformans* is strict, and leakage from the α- parent was approximately 5%, whereas at high temperature, almost 30% of the progeny inherited their mtDNA from the α-parent. When the Δ*atg8* strain was used as the *MAT***a-** and *MAT*α-parent, robust uniparental inheritance of mtDNA was observed with a slight increase in leakage from the α-parent (approximately less than 10%; Table 1). Almost the same result was obtained in the cross between Δ*atg7* strains (Table 1).

**Figure 4:**
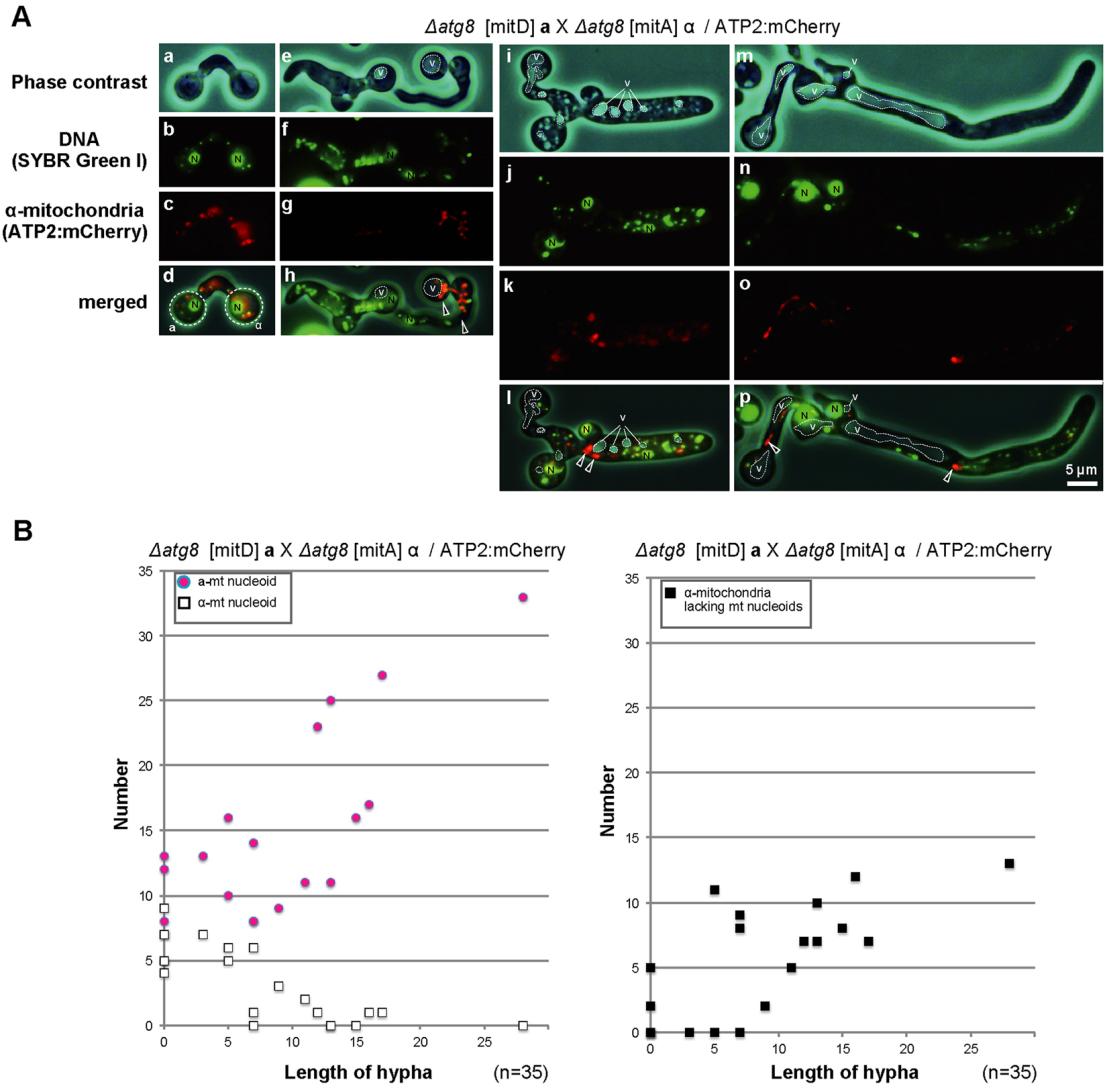
Diffusion of α-mitochondria lacking mt nucleoids in developing Δ*atg8* zygotes. (**A**) Phase contrast images (a,e,i,m), SYBR Green I signals (SYBR G: b,f,j,n), ATP2:mCherry signals (c,g,k,o), and merged images (d,h,l,p) of zygotes (Δ*atg8* [mitD] **a** XΔ*atg8* [mitA] *ATP2*:mCherry α) are shown. Mt nucleoids were detected in the early zygotes (d). Digestion of α-mt nucleoids occurred in the early stage of zygote development and α-mitochondria lacking mt nucleoids were detected throughout the cytoplasm, outside vacuoles (V), of zygotes [black arrowheads in (h,l,p)]. (**B**) The number of **a**- and α-mt nucleoids (left panel), and α-mitochondria lacking mt nucleoids (right panel) was plotted against the length of hypha in zygotes (Δ*atg8* [mitD] X Δ*atg8* [mitA]α/ATP2:mCherry). The number of zygotes analyzed was 35.

We observed the mating process of the Δ*atg8* strain under the microscope (Fig. 4). Normal filamentation was observed for the Δ*atg8* strain, indicating that loss of *ATG8* has no severe effect on cell viability or sexual development in *Cryptococcus* (Fig. S4). Under the microscope, the preferential reduction of α-mt nucleoids was seen to occur in early zygotes (Fig. 4). Interestingly, α-mitochondria lacking mt nucleoids were retained and diffused throughout the dikaryotic hyphae. As far as we investigated, all α-mitochondria were retained outside of vacuoles (in 35 zygotes), although their number declined as hyphae and vacuoles developed. Proliferation of **a**-mt nucleoids occurred in a similar manner as in WT (Figs. 4 and S1). These observations suggest that *ATG8* is responsible for the clearance of α-mitochondrial structures after the active digestion of α-mt nucleoids, which could explain the subtlety of the effect of Δ*atg8* on uniparental mtDNA inheritance (Table 1).

We next attempted to identify the DNase responsible for the preferential degradation. One candidate was a mitochondrial DNase, endonuclease G (*NUC1/EndoG*). In *C*. *elegance*, knockdown of CPS-6, mitochondrial endonuclease G, has been reported to impede the degradation of paternal mtDNA, leading to increased embryonic lethality^8^. Also, in the EndoG mutant of *Drosophila melanogaster*, the degradation of mtDNA is delayed, while there is a second mechanism to remove mtDNA from mature sperm^7^. To assess the function of *NUC1* in *Cryptococcus*-mtDNA inheritance, the *NUC1* homolog (CNE03360) was disrupted and mtDNA inheritance was investigated. *NUC1* deletion showed no significant effect on mtDNA inheritance (Table 1), nor did the double mutant Δ*atg8*Δ*nuc1* show enhanced disturbance of mtDNA inheritance, suggesting that *NUC1* is not involved in the regulation of mtDNA inheritance in *Cryptococcus*.

## Discussion

The mitochondrial genome is present as multiple copies per cell and is subject to dynamic regulation. Multiple copies of mtDNA or cpDNA are not simply present in an exposed state *in vivo*, but rather are compacted into DNA-protein complexes called nucleoids. mt nucleoids serve as sites for replication, recombination, and transcription, and they also function as units of segregation and inheritance^36^. In the present research, mt nucleoids were clearly visualized in living *C*. *neoformans* cells by using a dsDNA-specific fluorochrome, SYBR Green I, and their behaviors were monitored during zygote development (Fig. 1). Single cell analysis using optical tweezers was also performed to analyze the level of mtDNA in the zygotes (Fig. 2). The fate of α-mitochondrial structures was tracked using a membrane-voltage-dependent dye, Rhodamine123 (Fig. 3). Furthermore, using a gene disruption technique, the roles of autophagy and Endonuclease G (NUC1) in the uniparental inheritance were assessed (Fig. 4, Table 1).

**Figure 5:**
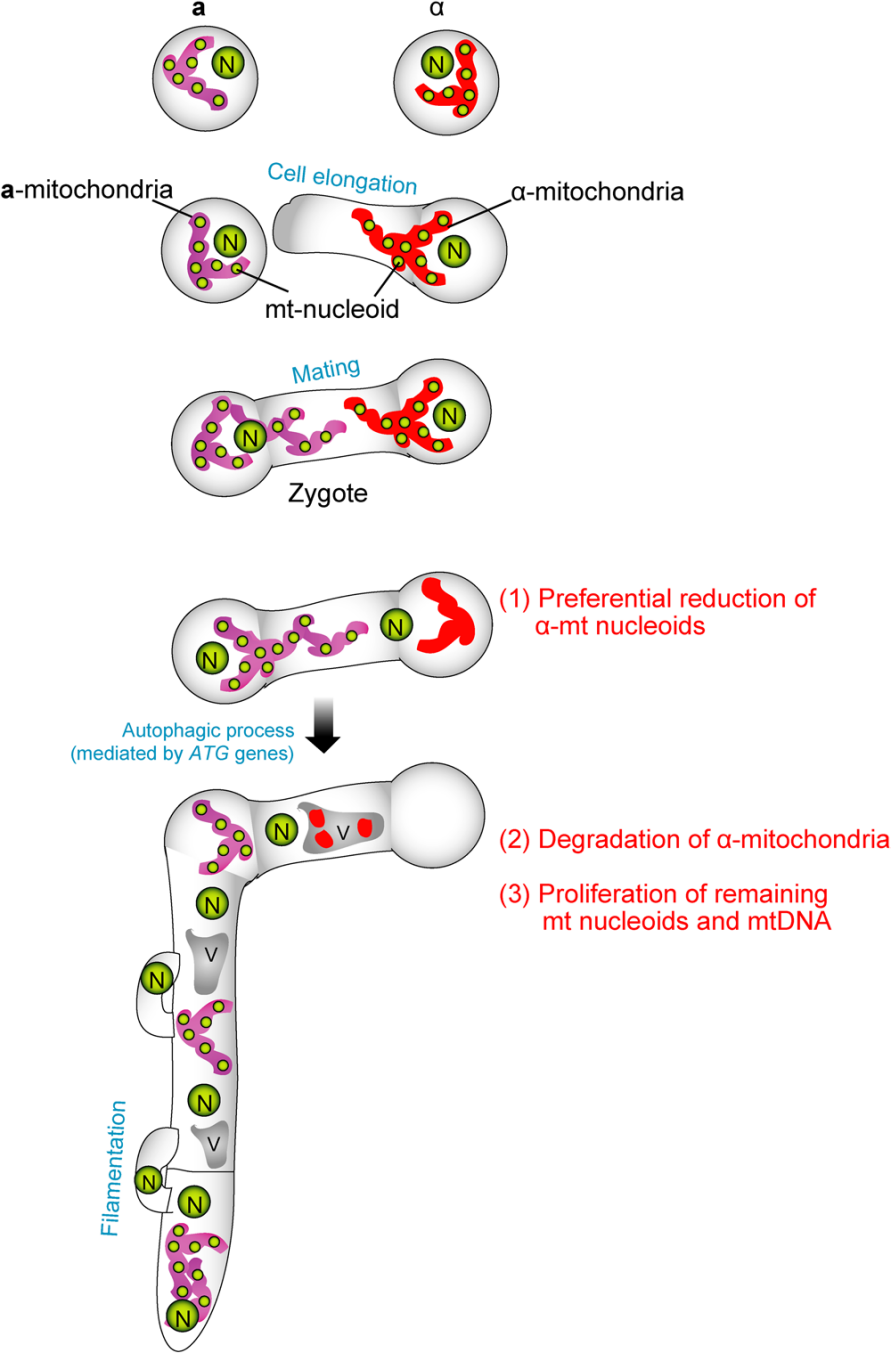
A schematic model explaining the three-step mechanism of uniparental inheritance of mtDNA in *Cryptococcus neoformans*. (1) Preferential reduction of α-mt nucleoids (2) Degradation of the α-mitochondrial structure and (3) Proliferation of remaining mitochondria.

Our analyses revealed that strict uniparental mitochondrial inheritance was achieved in three steps (summarized in Fig. 5): (1) preferential reduction of α-mt nucleoids (Figs. 1 and 2), (2) incorporation of α-mitochondria into vacuoles for further clearance (Fig. 3, Video S2, Fig. S2), and (3) proliferation of remaining mt nucleoids in zygotes (Figs. 1, 3 and S3). Notably, the preferential reduction of α-mt nucleoids was observed prior to the destruction of the α-mitochondrial structure (Fig. 1). This strategy would be advantageous to avoid leakage and diffusion of partially fragmented mtDNA molecules, which could trigger aberrant recombination, rearrangements and destabilization of mitochondrial genomes^37^. As for sperm mtDNA, it has been postulated that paternal mtDNA can be deleterious because it might be heavily damaged and accumulate mutations due to oxidative stresses^38^. In animals, leaked mtDNA could trigger inflammation^39^ by such mechanisms as causing production of second messengers cyclic GMP and cyclic AMP^40,41^. This process could be further stimulated when fragmented mtDNA molecules were bound by a mt nucleoid protein (transcription factor A (TFAM)^42^.

Presumably due to the preferential reduction of α-mt nucleoids, α-mitochondria were incorporated into vacuoles for further degradation (Fig. 4). Our analysis suggested that this process is probably mediated by autophagy, because disruption of the gene homologous to *ATG8* resulted in inefficient clearance of the α-mitochondrial structure (Fig. 4), whereas it did not affect the preferential reduction of α-mt nucleoids (Fig. 4) or the mode of uniparental inheritance (Table 1). This result indicates that the degradation of the mitochondrial structure may be the final step in the elimination of α-mitochondria. Furthermore, it suggests that the reduction of α-mtDNA is independent of autophagy and is the critical process that determines the uniparental inheritance of mtDNA in *C*. *neoformans*.

The actual mechanism underlying the preferential elimination of mtDNA remains unclear. In *C*. *neoformans*, it is unlikely that the gene homologous to endonuclease G is involved in the regulation of mtDNA inheritance (Table 1). There may be other still unknown DNase(s) responsible for this process.

Another possibility is that *de novo* synthesis of α-mtDNA is specifically blocked, leading to the elimination/dilution of α-mtDNA via constitutive turnover of mtDNA. In our EdU labeling analysis, however, the intensity of EdU signals was not significantly different between **a**- and α-mitochondria in haploid cells (gametes) and early zygotes, and we could not find solid evidence to support the possibility of specific inhibition of α-mtDNA synthesis or preferential replication **a**-mtDNA (Fig. S3). Meanwhile, clear EdU signals were detected from **a**-mitochondria in developing hypha, indicating the occurrence of **a**-mtDNA replication, which may contribute to the dilution of residual α-mtDNA. Further optimization of the experimental conditions, such as synchronization of the mating reaction and minimizing the EdU-labelling time would be necessary to assess the precise role of mtDNA replication in the uniparental inheritance (Fig. S3).

In *C*. *neoformans*, the downstream events as well as the regulatory mechanisms of uniparental inheritance and the mating process have been extensively studied^32^. Mating-type-specific homeotic proteins, Sxi2**a** and Sxi1α, regulate uniparental inheritance in cooperation with the transcription factor Mat2. Coincidentally, it was found that uniparental inheritance in *Chlamydomonas*, which is evolutionarily divergent from *C*. *neoformans*, is also regulated by a mating-type-specific homeotic gene, *GAMETE SPECIFIC PLUS* (*GSP*)*1*^18^. Detailed investigation of the downstream genes regulated by these homeotic proteins^43^ will be important for elucidating the mechanisms of the uniparental inheritance.

Understanding the molecular basis of uniparental inheritance would open up the way to regulate mtDNA inheritance. In human mitochondrial diseases, wild-type and mutant alleles coexist (heteroplasmy), and a higher extent of heteroplasmy causes various severe symptoms^44^. In humans, a woman with a deleterious heteroplasmic mtDNA mutation may pass the mutation to her offspring. To avoid the transmission of such deleterious mtDNA, transplantation of mitochondria from another healthy parent has been successfully applied to produce a so-called “three-parent baby”^45^. The manipulation of paternal transmission of mtDNA would be another potential cure. Recently, multi-generation biparental transmission of mtDNA in humans has been reported^46^, suggesting the existence of genetic mechanisms controlling the maternal inheritance and the possibility of manipulating mtDNA inheritance without affecting other fertilization processes or viability.

The mechanism of the inheritance of mitochondrial and chloroplast DNA may vary among species^6^. Recently, an unexpected and interesting possibility that lipid biosynthesis would be key to determining the competition of cpDNA inheritance has been proposed in evening primroses^47^. Further analyses to identify the gene(s) responsible for the uniparental inheritance of mtDNA in *C*. *neoformans* would shed light on the diversity or universality of mechanisms underlying uniparental inheritance.

## Methods

### Strains and growth conditions

Strains used in this study are listed in Supporting Information, Table S1. All of the strains used in this study were derivatives of *C*. *neoformans* JEC21 (*MAT*α serotype D [mitD]). mitA Mitochondria were transferred from a serotype A strain (CAT1039, *MAT***a** *ura5* derivative of NK99a), into a serotype D strain, TLHM15 (*MAT*α), by isolation of blastospores produced during mating between CAT1039 and TLHM15. Yeast cells were grown and maintained on YPD medium (1% yeast extract, 2% Bacto peptone, and 2% dextrose). Mating of *C*. *neoformans* was conducted on hay-cube (HC) medium (pH 6.2) at 22 °C. Equal numbers of **a-** and α-cells were collected using a toothpick and were thoroughly mixed in 50 µL distilled water (DW); 450 µL DW was added to each cell suspension, and 5 µL of each suspension was spotted and dried on an HC agar plate.

### Fluorescence microscopy

Vital staining of DNA was performed by adding SYBR Green I (Invitrogen, Carlsbad CA, USA) at 1/2000 dilution. To visualize mitochondria, Rhodamine 123 (Invitrogen) was added to cell suspensions at a final concentration of 0.2 µg/mL and the stained cells were incubated for approximately 10 min at room temperature. To stain vacuoles, yeast vacuole membrane marker MDY-64 (Yeast vacuole marker sampler kit, Invitrogen) was used at the concentration of 10 µM. The stained cells were observed with a fluorescence/phase contrast microscope (Axiovert 135; Zeiss, Oberkochen, Germany) connected to a cooled charge-coupled device color camera (DP73; Olympus, Tokyo, Japan).

### Optical tweezers and PCR analysis of mtDNA

The configuration of the optical tweezers was described in our previous report^20^. To manipulate very sticky filamentous zygotes with optical tweezers, a buffer containing 1% BSA and 0.05% Triton X was used. Individual cells were observed under a fluorescence microscope, trapped with the optical tweezers, and harvested into PCR tubes as previously described (Fig. S2, Movie S1)^17,20^.

Single-nucleotide polymorphisms between serotypes A and D were used to distinguish **a** and α-derived mtDNAs. The individual zygotes obtained with the optical tweezers were subjected to nested PCR for the mitochondrial *nd5* gene using the following conditions: first amplification, YN732_Cn_nd5_F0 + YN733_Cn_nd5_R0 primers, and second amplification, YN734_Cn_nd5_F1 (dCAPS: PvuII in A) + YN735_Cn_nd5_R1 (151 bp: 127 bp + 24 bp after PvuII digestion) or YN736_Cn_nd5_F2 (dCAPS: BglII in D) + YN737_Cn_nd5_R2 (132 bp: 110 bp + 22 bp after BglII digestion) primers. dCAPs primers were designed using dCAPs Finder 2.0 (http://helix.wustl.edu/dcaps/). The PCR products were digested using PvuII or BglII and separated by electrophoresis on a 4% NuSieve 3:1 agarose gel.

We also designed a primer pair (YN774_Cn_nd5_F3 + YN775_Cn_nd5_R3) that amplified a 400-bp fragment from mtDNA of serotype A and a 1.4-kb fragment from serotype D.

### Colony PCR

Colony PCR was performed using a protocol reported for yeast. Cells were suspended in 1 mg/mL Zymolyase 100 T and incubated for 5 minutes at 37 °C. The cell suspensions were vortexed and 1 µL of the supernatant was used for colony PCR with GoTaq (Promega).

### EdU labelling of zygotes

EdU labeling was performed using a kit (Click-iT Plus Alexa488 imaging kit, Thermo Fisher Scientific, Waltham, MA USA) and following a protocol reported for *S*. *cereviciae*^35^. Mating of *C*. *neoformans* was induced on hay cube agar plates, on which 200 µL of 1 mM EdU (5′-ethynyl-2′-deoxyuridine) was spread and dried. To enhance the incorporation of EdU, 200 µL of 0.1 mM FdUrd (5′-fluoro-2′-deoxyuridine: Nacalai Tesque, Kyoto, Japan), which is an inhibitor of thymidine synthase, was applied on the agar plates, following a protocol for BrdU (5′-bromo-2′-deoxyuridine) labeling^48^. After 8 hrs, cells were harvested from the agar plates, suspended in 1X PBS (pH 7.5) and fixed with 2% freshly-prepared PFA (paraformaldehyde) for 20 min, permeabilized in 70% (vol/vol) ethanol for 3 hr, incubated in 1X PBS with 3% BSA (bovine-serum-albumin) for 30 min; washed twice with 1X PBS with 3% BSA, and incubated in 200 uL of Click-iT EdU Alexa Fluor 488 reaction mix for 30 min. Cells were then washed twice with 1X PBS with 3% BSA; mixed with DAPI (final concentration 1 µg/mL) and observed under a fluorescence microscope.

### Construction and transformation of *ATP2*:mCherry and gene disruption of *ATG8* and *NUC1*

We constructed the mCherry-*URA5* cassette using a PCR method. The 3′ 1-kb DNA fragment of *ATP2* was amplified by PCR using primers 1471 and 1472 and genomic DNA of JEC21 as the template. An *ATP2*:mCherry fusion was constructed with overlapping PCR using primers 1471 and 997 and the above 1-kb DNA fragment and mCherry-*URA5* cassette as templates. The resulting fusion product contained the *ATP2*:mCherry fusion and half of *URA5*. The 1-kb segment downstream of the stop codon of *ATP2* was amplified by PCR using primers 1473 and 1474 and genomic DNA as template. This fragment was fused to the other half of *URA5*. The two fusion fragments, sharing 200 bp at the truncated *URA5* end, were used as donors for biolistic transformation (using the split marker method).

A standard overlap PCR approach was used to disrupt *ATG8* and *NUC1* in the strains of CAT250**a** with *URA5* and hygromycin-resistance markers, respectively. Primers used for gene disruption are listed in Table S2. The overlap PCR products were introduced into the genome using the biolistic transformation system IDERA GIE-III (Tanaka Co. Ltd., Sapporo, Japan).

## Supplementary information

Supplemental information.

Video S1.

Video S2.
