# Supplementary material for: Step-wise elimination of α-mitochondrial nucleoids and mitochondrial structure as a basis for the strict uniparental inheritance in Cryptococcus neoformans
Source: Sci Rep. 2020 Feb 12;10:2468. doi: 10.1038/s41598-020-59277-9 (PMC7016115; doi:10.1038/s41598-020-59277-9)
Supplement: Supplementary file 1 — Supplemental information. [file 41598_2020_59277_MOESM1_ESM.pdf]

Supplemental information for

Step-wise elimination of α-mitochondrial nucleoids and mitochondrial structure as a basis for the strict uniparental inheritance in *Cryptococcus neoformans*

Yoshiki Nishimura[1*], Toshiharu Shikanai[1], Susumu Kawamoto[2], and Akio Toh-e[2]

[1]Department of Botany, Kyoto University, Kita-Shirakawa, Oiwake-cho, Kyoto 606-8502, Japan

[2]Division of Clinical Research, Medical Mycology Research Center, Chiba University, 1-8-1 Inohana, Chuo-ku, Chiba 260-8673, Japan

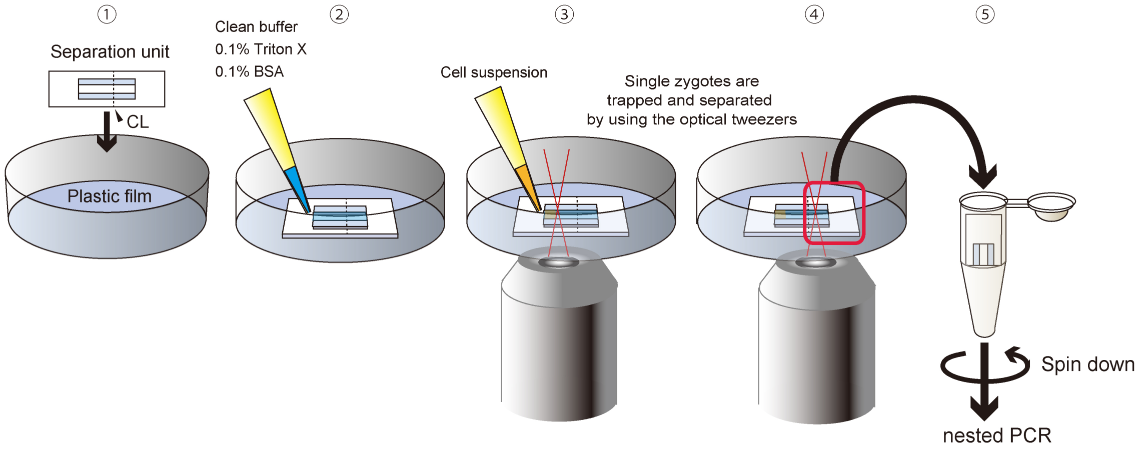

Figure S1 Strategy for isolating a single cell using optical tweezers.

(1) A plastic dish with a plastic film bottom is prepared. One droplet of distilled water (DW) is placed on the film. A "separation unit" with a cutting line (CL) is placed on the droplet so that it adheres closely to the plastic film. (2) The microchamber of the separation unit is filled with sterile buffer (0.1% Triton X, 0.1% BSA). (3) A cell suspension is added to one end of the microchamber. (4) A single cell of interest is targeted, trapped, and transferred to the other end of the microchamber using optical tweezers. The end containing the cell of interest is cut at the CL and inserted into a PCR tube. The cell is spun down in the PCR tube by centrifugation.

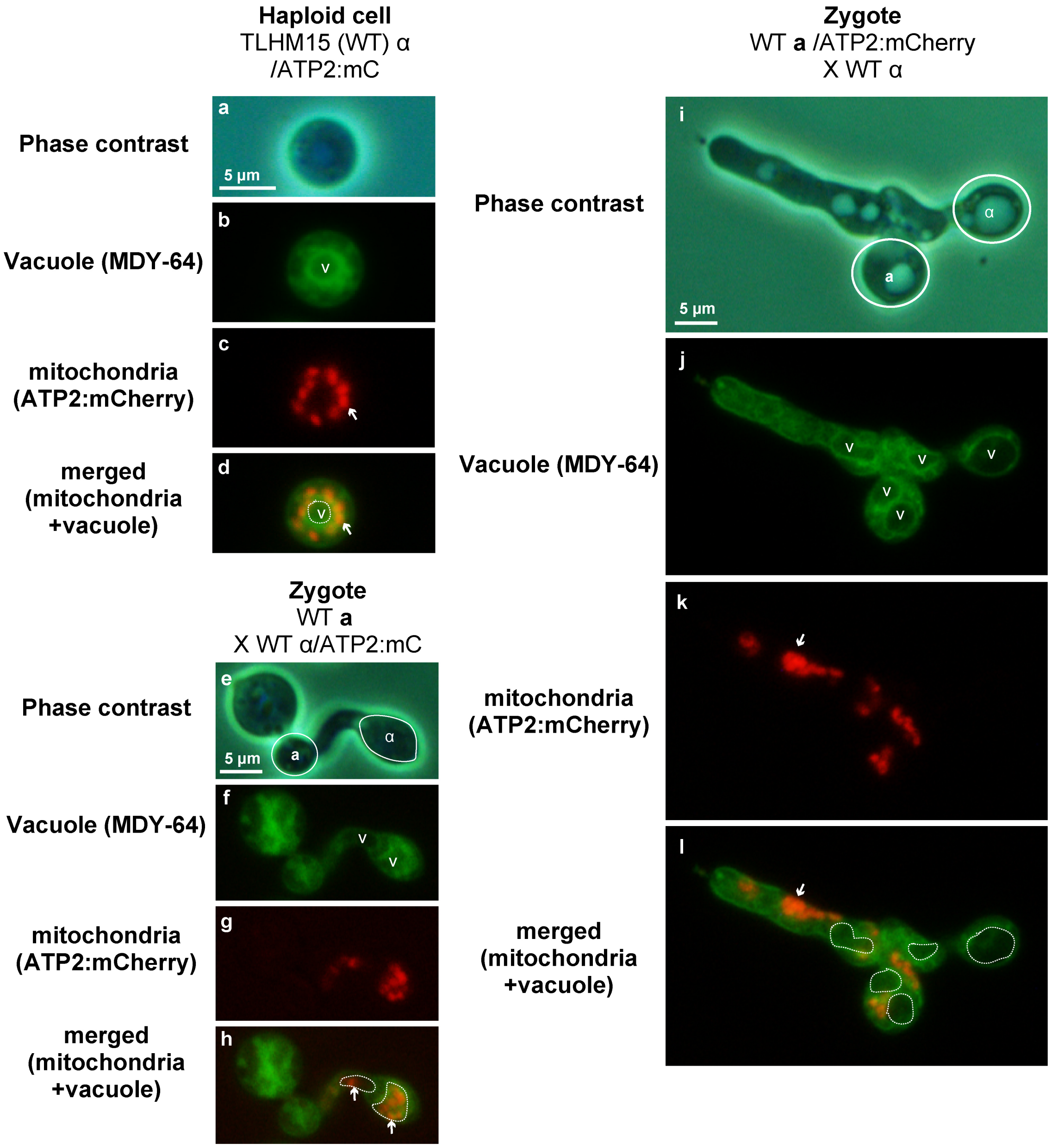

Figure S2 Vacuoles in haploid cells and zygotes.

Vacuoles in haploid cells (a-d), zygote (WT a X WT α/ATP2:mCherry)(e-h), and zygote (WT **a**/ATP2:mCherry X WT α) (i-l) were stained for a vacuole marker (MDY-64). Phase contrast (a, e, i), MDY-64 (b, f, j), ATP2:mCherry (c, g, k) and merged images showing MDY-64 and mCherry signals (d, h, l). The positions of **a**- and α- parental cells in zygotes are indicated by circles in e and i. mCherry-labelled mitochondria are indicated by arrows in c, d, h, k, l. V: vacuole. 29 zygotes were analyzed.

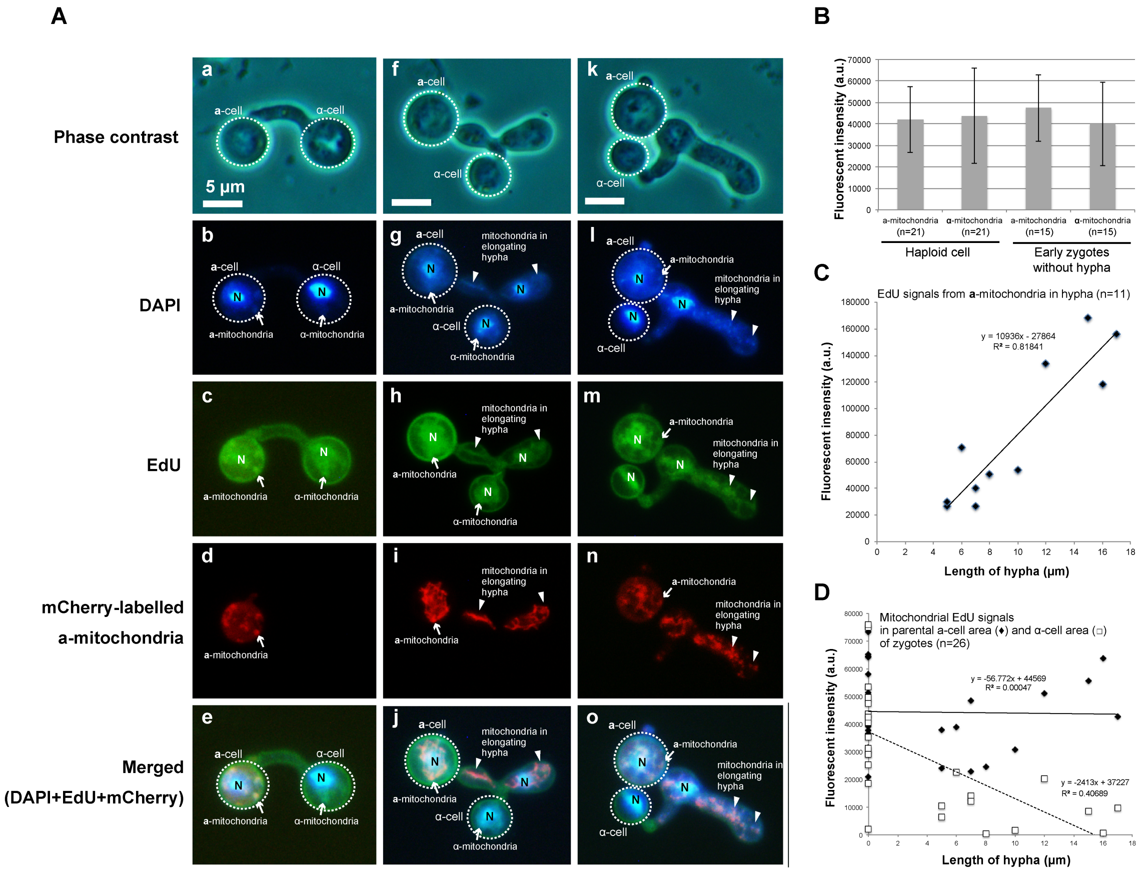

Figure S3 mtDNA sysnthesis in zygotes analyzed by EdU labelling.

(A) mtDNA sysnthesis in zygotes was analyzed by EdU labelling. Phase contrast (a, f, k), DAPI (b, g, l), EdU (c, h,m), and ATP2:mCherry-labelled **a**-mitochondria (d, i, n), and merged images (DAPI+EdU+ATP2:mCherry) (e, j, o) are shown. The areas of **a**- and α- parental cells are encircled by dotted lines. N: cell nucleus. DNA synthesis in the cell nucleus (N) and in mitochondria (arrows) was detected by EdU labelling. Notably, a-mitochondria in elongating hypha were clearly labelled by EdU (arrowheads in h, m), indicating that the proliferation of a-mitochondria in zygotes is probably accompnanied by mtDNA synthesis. (B) The fluorescence intensity of mitochondrial EdU signal (in a.u.: arbitrary unit) was compared between **a**- and alpha- mitochondria in haploid cells and early zygotes. No significant difference was detected. (C) The fluorescence intensity of **a**-mitochondrial EdU signal in elongating hypha (◆). An approximate straight line and its equation are indicated. (D) The fluorescence intensity of mitochondrial EdU signal in the parental **a**-cell area (◆) and α-cell area (□).Approximate straight lines (solid line for **a**-cell and dashed line for α-cell areas) and their equations are shown.

YN15_22**a** *Δatg8* [mitD]
X
YN15_6α *Δatg8* [mitA] / ATP2:mCherry

CAT1209**a** *Δatg8* Δ*nuc1* [mitD]
X
CAT1086α *Δatg8* Δ*nuc1* [mitA] / ATP2:mCherry

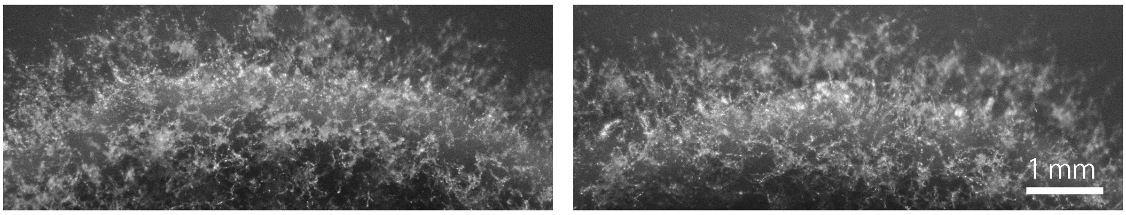

Figure S4 Filamentation of *Δatg8* and *Δnuc1* mutants.

Normal filamentation after mating was observed for *Δatg8* and *Δnuc1* mutants.

Strain	Genotype
CAT250（WT）	*MAT***a** *ura5 str2*::*URA5* [mtD]
TLHM15（WT）	*MAT*α *ura5 cku70*::*NEO* [mtD]
CAT1072	*MAT***a** *ura5 str2*::*URA5* [mtA]
CAT1073	*MAT*α *ura5 str2*::*URA5* [mtA]
CAT1022	*MAT***a** *ura5 ATP2*:mCherry-*URA5* [mtD]
CAT1023	*MAT*α *ura5 ATP2*:mCherry -*URA5* [mtD]
CAT1097	*MAT***a** *ura5 atg8::URA5* [mtA]
YN15_22	*MAT***a** *ura5 atg8::URA5* [mtD]
YN15_6	*MAT*α *ura5 atg8::URA5* ATP2:mCherry-*URA5* [mtA]
CAT1079	*MAT*α*ura5 nuc1::HYG* [mtD]
CAT1054	*MAT***a** *ura5 nuc1::HYG ATP2*:mCherry-*URA5* [mtD]
CAT1080	*MAT***a** *ura5 nuc1::HYG* [mtD]
CAT1055	*MAT*α *ura5 nuc1::HYG ATP2*:mCherry-*URA5* [mtD]
YN16_12	*MAT*α *nuc1::HYG* ATP2:mCherry-*URA5* [mtA]
CAT1086	*MAT*α *ura5 atg8::URA5 nuc1::HYG ATP2*:mCherry -*URA5* [mtA]
CAT1087	*MAT*α *ura5 atg8::URA5 nuc1::HYG* [mtA]
CAT1208	*MAT*α *ura5 atg8::URA5 nuc1::HYG* [mtD]
CAT1209	*MAT***a** *ura5 atg8::URA5 nuc1::HYG* [mtD]
YN3_6	*MAT*α *ura5 atg8::URA5 nuc1::HYG ATP2*:mCherry-*URA5* [mtD]
YN4_9	*MAT***a** *ura5 atg8::UTA5 ATP2*:mCherry-*URA5* [mtA]
YN4_11	*MAT*α *ura5 atg8::URA5* [mtA]
YN4_1	*MAT***a** *ura5 atg8::URA5 nuc1::HYG ATP2*:mCherry-*URA5* [mtA]
YN4_14	*MAT*α *ura5 atg8::URA5 nuc1::HYG ATP2*:mCherry-*URA5* [mtA]
YN4_15	*MAT***a** *ura5 atg8::URA5 nuc1::HYG* [mtA]

Table S1 Strains used in this study.

Primer #	Name	Sequence	Note
**Disruption of *ATG8* and *NUC1***			
1488	ATG8-Fl1-F	ACGAGTTCAGAGGAGGGGAATTTG	Primers to amplify the upstream of *ATG8*. Lower case: *URA*5 cassette sequence.
1489	ATG8-Fl1-R	actggccgtcgttttacaacgtcgtgactgggaaaaccctggcgGTTGCTATTGAAGTGGGTGGGATGTG	
1490	ATG8-Fl2-F	taatcatggtcatagctgtttcctgtgtgaaattgttatccgcCGGCAAAAGGACGCTAAAGGAACGG	Primers to amplify the downstream of *ATG8*. Lower case: *URA*5 cassette sequence.
1491	ATG8-Fl2-R	TACACCTTGCTCGTTGCATTTGACC	
	M13_R	CAGGAAACAGCTATGA	Primers to amplify 5' segment of *URA5*
998	URA5_R	TCATCGATGATGACGATTCG	
997	URA5_F	TACAAGGGTATCTCCTTGGC	Primers to amplify 3' segment of *URA5*
	M13_F	GTAAAACGACGGCCAG	
1168	NUC1-Fl1-F	TTCAGATATATGTGACGGTCGC	
1169	NUC1-Fl1-R	taatcatggtcatagctgtttcctgtgtgaaattgttatccgctTTTGGGAGTGGAGAGTGACTGG	
1170	NUC1-Fl2-F	ccaaaaaacacacgatcatcttcatactcccctctcaaagATGCTCCTCCCAAGGAAAACCTC	
1171	NUC1-Fl2-R	GATTATCACCGCCAAGAACACTCA	
**Colony PCR primers for genotyping progeny**			
YN764	MATa_F(STE20aD)	GTTCATCAGATACAGAGGAGTGG	MATa specific primer
YN765	MATa_R(STE20aD)	CTCCACTGTCAAACCTACGGC	
YN762	MATα_F(STE20aD)	ATAGGCTGGTGCTGTGAATTAAG	MATα specific primer
YN763	MATα_R(STE20aD)	GTTCAAGTAATCTCACTACATGCG	
YN758	Hyg_F	GAGCCTGACCTATTGCATCTC	Primers to detect Hygromycin resistant marker
YN759	Hyg_R	GATGTTGGCGACCTCGTAT	
YN766	ATG8_F (=1488)	ACGAGTTCAGAGGAGGGGAATTTG	Primers to detect the chimeric sequence *ATG8-URA5* (reversed)
YN760	URA_F	CTTCAATGCCGGTCTCCTTTA	
YN774	nd5_F3	CTATTGGTGTTACAGGAGCTCAC	Primers to distinguish mtDNA of serotype A (400 bp) and serotype D (1.4 kb)
YN775	nd5_R3	GAGCCTTCATACCTGCCTTATTTGC	
**dCAPs primers to distinguish mtDNA of serotype A and D**			
YN732	Cn_nd5_F0	ATGGGATATCTATTTATGGCAGTAG	
YN733	Cn_nd5_R0	TAATTGTAATACTTGGTACTACATG	
YN734	Cn_nd5_F1 (PvuII in A)	TCTTTAAGGCTCTACTATTCCCAGC	dCAPs primer sets to create PvuII site in mtDNA of serotype A
YN735	Cn_nd5_R1	GGTAGTGCCATTAGTGATAGTGATC	
YN736	Cn_nd5_F2 (BglII in D)	GTTGGTACTGATTTCCTATCAAGA	dCAPs primer sets to create BglII site in mtDNA of serotype D
YN737	Cn_nd5_R2	AGTGCTAGACCTGCACCAAATAGAC	
**Construction of *ATP2*-mCherry chimeric gene**			
1471	ATP2-mC-Fl1-F	TCAGCACTTGGGTGAGAACACTG	
1472	ATP2-mC-Fl1-R	CTTGATGATGGCCATGTTATCCTCCTCGCCCTTGCTCACCATagcaccctgctccttgagagacttc	
1473	URA5-ATP2-Fl2-F	TCATAAGGAACGCAAACGTATATCACCCATGCCTATCCGATggtgtcaggtgggatagtgtagaa	
1474	URA5-ATP2-Fl2-R	GTTTCCACATGACCTCGAAATCAC	

Table S2 Primers used in this study.

Video S1 Process of trapping and transferring a single zygote using optical tweezers. The cell is transferred from the area populated with unmated cells (left) to an area free of contamination with other cells (right). The video is in real-time.

Video S2 α-mitochondria incorporated into vacuoles as ATP2:mCherry-labelled particles showing Brownian motion. The video is in real-time.

Electrophoretic gel images

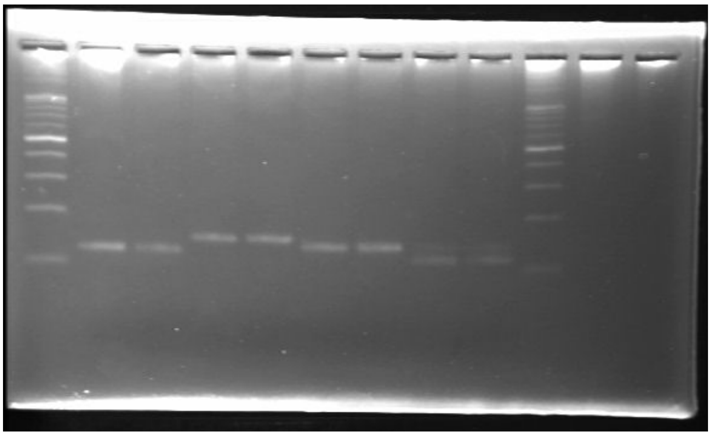

Electrophoretic gel image for Figure 3B

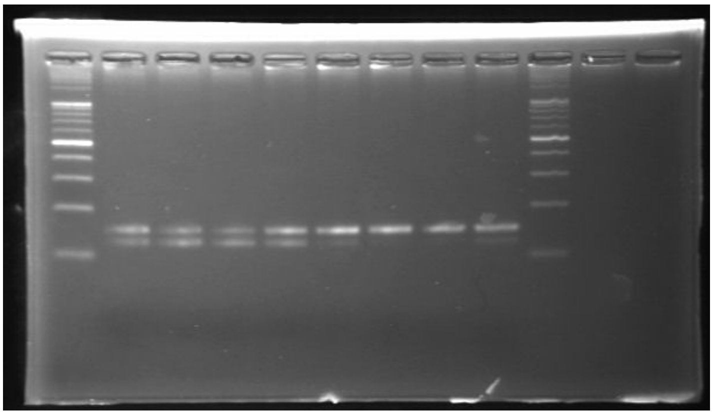

Electrophoretic gel image for Figure 3C

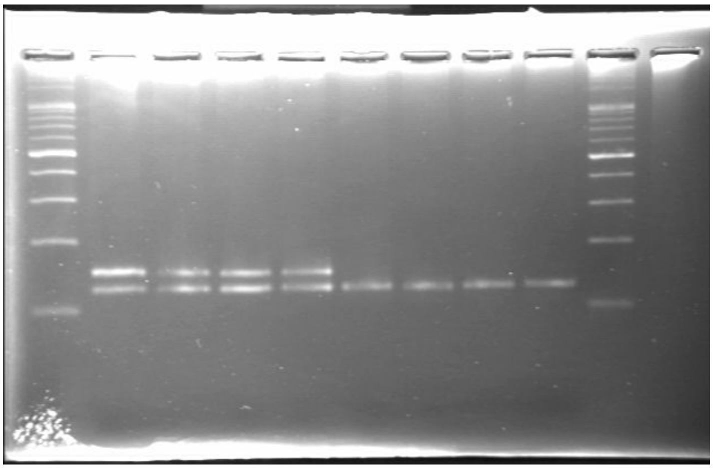

Electrophoretic gel image for Figure 3D

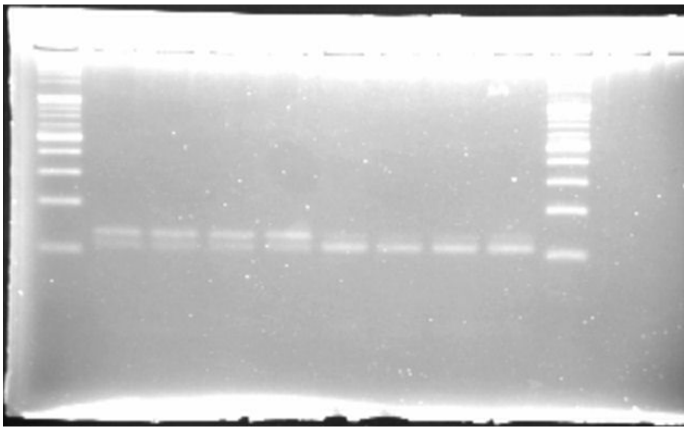

Electrophoretic gel image for Figure 3E

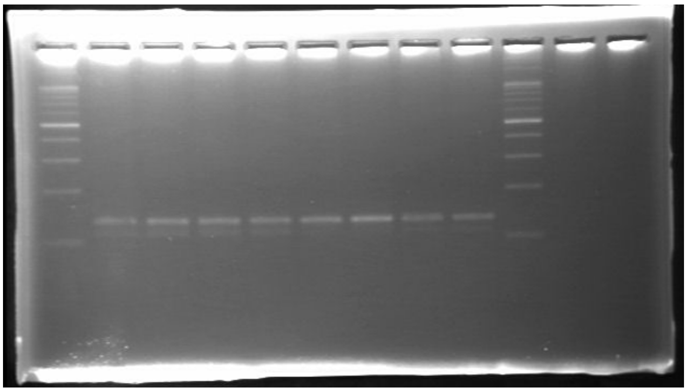

Electrophoretic gel image for Figure 3F

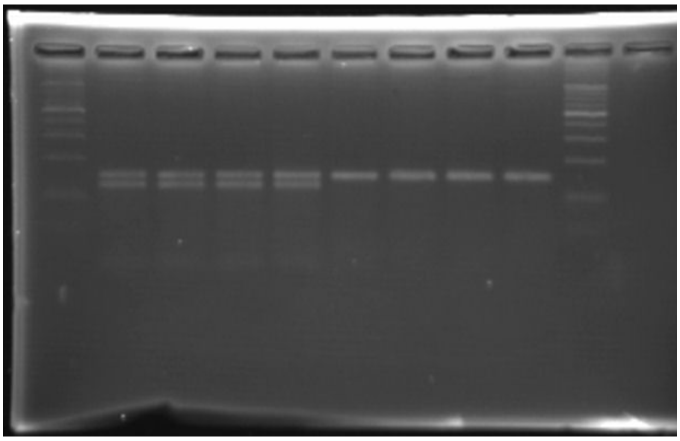

Electrophoretic gel image for Figure 3G

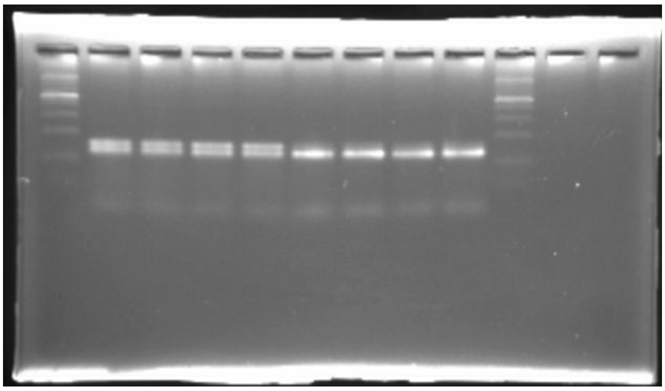

Electrophoretic gel image for Figure 3H
